# Oligodendrocyte Response to Pathophysiological Conditions Triggered by Episode of Perinatal Hypoxia-Ischemia: Role of IGF-1 Secretion by Glial Cells

**DOI:** 10.1007/s12035-020-02015-z

**Published:** 2020-07-21

**Authors:** Justyna Janowska, Justyna Gargas, Malgorzata Ziemka-Nalecz, Teresa Zalewska, Joanna Sypecka

**Affiliations:** NeuroRepair Department, Mossakowski Medical Research Centre, Polish Academy of Sciences, 5, A. Pawinskiego Str., 02-106 Warsaw, Poland

**Keywords:** Glial cells, Oligodendrocyte maturation, Astrocytes, Microglia, Neural development, Perinatal asphyxia, Neonatal hypoxia-ischemia, IGF-1 secretion, Autocrine/paracrine effect, Sholl analysis of cell branching

## Abstract

Differentiation of oligodendrocyte progenitors towards myelinating cells is influenced by a plethora of exogenous instructive signals. Insulin-like growth factor 1 (IGF-1) is one of the major factors regulating cell survival, proliferation, and maturation. Recently, there is an ever growing recognition concerning the role of autocrine/paracrine IGF-1 signaling in brain development and metabolism. Since oligodendrocyte functioning is altered after the neonatal hypoxic-ischemic (HI) insult, a question arises if the injury exerts any influence on the IGF-1 secreted by neural cells and how possibly the change in IGF-1 concentration affects oligodendrocyte growth. To quantify the secretory activity of neonatal glial cells, the step-wise approach by sequentially using the in vivo, ex vivo, and in vitro models of perinatal asphyxia was applied. A comparison of the results of in vivo and ex vivo studies allowed evaluating the role of autocrine/paracrine IGF-1 signaling. Accordingly, astroglia were indicated to be the main local source of IGF-1 in the developing brain, and the factor secretion was shown to be significantly upregulated during the first 24 h after the hypoxic-ischemic insult. And conversely, the IGF-1 amounts released by oligodendrocytes and microglia significantly decreased. A morphometric examination of oligodendrocyte differentiation by means of the Sholl analysis showed that the treatment with low IGF-1 doses markedly improved the branching of oligodendroglial cell processes and, in this way, promoted their differentiation. The changes in the IGF-1 amounts in the nervous tissue after HI might contribute to the resulting white matter disorders, observed in newborn children who experienced perinatal asphyxia. Pharmacological modulation of IGF-1 secretion by neural cells could be reasonable solution in studies aimed at searching for therapies alleviating the consequences of perinatal asphyxia.

## Introduction

To acquire the ability to myelinate the central nervous system (CNS), oligodendrocyte progenitor cells (OPCs, so called NG2-glia) have to undergo a multistage differentiation process, which is guided by a plethora of extracellular instructive signals. Some of them are known to guide OPCs migration, like for instance the activity of metalloproteinases which help to reorganize the extracellular matrix and facilitate cell trafficking, the gradient of PDGF-AA concentration in the local microenvironment, as well as the presence of either chemoattractants or chemorepellents associated with normal or pathophysiological conditions. Other signaling molecules are known to be engaged in cell survival, proliferation, and initiation of myelin gene expression [1]. Finally, the multibranched mature oligodendrocytes are able to extent their unique, specialized cell processes and to wrap them around axonal segments forming multilamellar, tightly compacted myelin sheaths [2–4].

One of the major factors shown to regulate oligodendrocyte functions is the insulin-like growth factor-1 (IGF-1), distributed throughout the body by circulating blood, but also secreted in situ in the nervous tissue [5]. This small, a 7.64-kDa peptide shares many similarities with insulin, including high sequence analogy and common signal transduction pathways. Accordingly, the IGF-1 acts through the canonical extracellular-regulated kinase (ERK) and phosphatidylinositol-3 kinase (PI3K)-Akt pathways, as well as through the JAK/STAT signaling cascade [6–10]. This growth factor is thought to be essential for normal brain development [11], by promoting neurogenesis, elongation of neuronal projections, dendritic arborization, and synaptogenesis [12–16]. In the nervous tissue, IGF-1 has been shown to serve also as a neuroprotectant, promoting neuronal survival, and proliferation [17–21]. Thus, it is hypothesized that in certain pathophysiological conditions occurring in the CNS (like for instance stroke, infections, autoimmunological diseases, hypoxic-ischemic episodes), the availability of this factor and the sensitivity of cells to its influence in various brain regions might be one of discriminative factors between the onset of neurodegenerative disorders and capability to overcome the local tissue crisis [22–26]. Accordingly, alterations in the IGF-1 level are supposed to be associated with the development of white matter diseases, resulting from myelin deficiency or malformation and subsequent white matter disorganization. And indeed, a growing list of evidence indicates that the IGF-1 plays an important role in controlling oligodendroglial functions, including promotion of developmental myelinogenesis [27]. Although the alterations in the IGF-1 concentration are thought to be associated with the fatal consequences of white matter disorders developing as a result of hypoxic-ischemic insult experienced by newborn children [28], the exact mechanism of pathogenesis remains still largely unknown.

Likewise, IGF-1 is supposed to be involved also in subsequent stages of oligogliogenesis and myelinogenesis. Likewise, it has been shown to stimulate the glial commitment of neural stem cells [29–31], to enhance rate of OPC proliferation [32–37], to promote their survival [38], and to direct their migration by activation of integrin-mediated intracellular signaling [39]. During the middle stages of oligodendrocyte development, IGF-1 regulates protein synthesis through the PI3K/mTOR/Akt and MEK/ERK pathways contributing to the progress in differentiation process [40–43]. Finally, this growth factor is engaged in initiation and coordination of myelinogenesis, as well as has been shown to promote remyelination [27, 44–48]. Taking into consideration its well established role in the neurogenesis and brain development [49, 50], maintaining the physiological level of IGF-1 seems to be crucial for local tissue homeostasis and, thus, for proper CNS development and functioning.

**Fig. 1:**
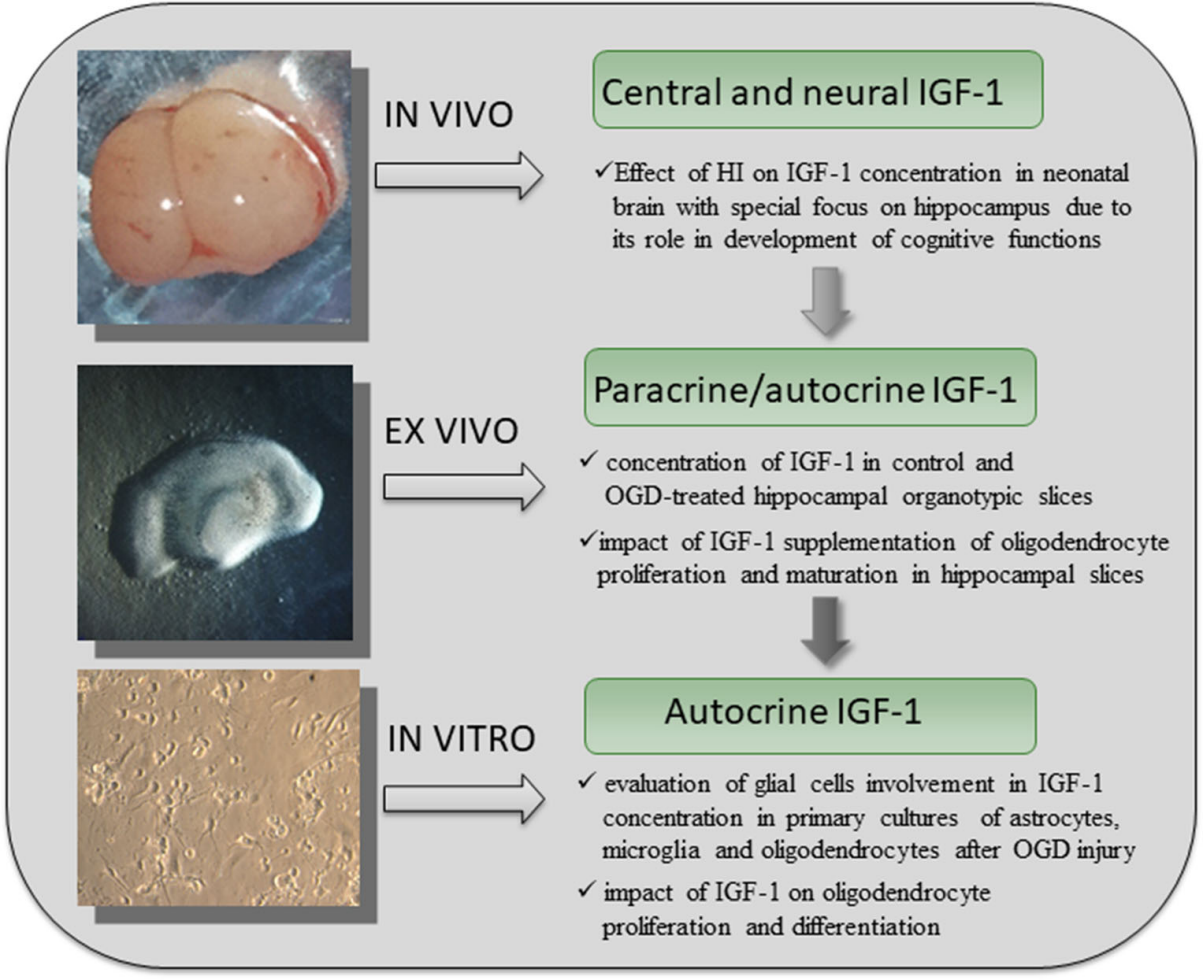
A schematic diagram of a step-wise approach aimed at determining the role of the IGF-1 released in situ by glial cells inhabiting the CNS

To address this issue, a study investigating the impact of temporal hypoxia-ischemia on IGF-1 secretion by particular subpopulations of CNS glia was designed, based on the in vitro (glial primary cell monocultures), ex vivo (hippocampal organotypic slice culture), and in vivo (rat model of perinatal asphyxia) experiments (Fig. 1). This approach allowed us to determine the IGF-1 quantities after perinatal asphyxic insult in vivo and to take a closer look at its secretion by the specialized glial cells in vitro. We hypothesized that potential alterations in the level of centrally distributed IGF-1, triggered by neonatal hypoxia-ischemia might exert an acute effect on oligodendroglial cells and aimed at evaluating a role of paracrine/autocrine IGF-1 signaling in neonatal brains. Thus, the obtained results allowed us to speculate about contribution of the glial cells to the pathogenesis of perinatal asphyxia and indicate potential therapeutic strategies.

## Materials and Methods

### Rat Model of Neonatal Hypoxia-Ischemia

To evoke the hypoxic-ischemic (HI) insult in animal model of perinatal asphyxia, Wistar rat pups (*n =* 48) of both sexes (12–15 g body weight) were anaesthetized with isoflurane (4% for induction of anesthesia and 2% for anesthesia maintenance) on 7 postnatal day (P7). The procedure, approved by IV Local Ethics Committee on Animal Care and Use, was based on the dissection of the left common carotid artery, which was either exposed (sham-operated animals, *n =* 20) or cut between the double ligatures of silk sutures (*n =* 28). After treating the resulting wound with lignocaine, animals were allowed to recover for 60 min in their home cages. Hypoxic conditions were achieved by exposing the animals to 7.5% oxygen in nitrogen for 60 min in a hypoxic chamber heated to 35 °C. Consequently, the hemisphere ipsilateral to the carotid ligation experienced the ischemic-hypoxic injury.

### Ex Vivo Culture of Organotypic Hippocampal Slices

The hippocampi for preparation of 400-μm thick organotypic slices with the preserved tissue organization were isolated from the brains of 7-day-old Wistar rats (*n* = 18), according to the protocol described previously [51]. The procedure was approved by the IV Local Ethics Committee on Animal Care and Use (Ministry of Science and Higher Education). The slices, obtained by cutting the chilled hippocampi by use of a McIlwain apparatus, were placed on Millicell-CM (Millipore) membranes and cultured initially in DMEM medium (Gibco) containing the following supplements: 25% horse serum (Gibco), 25% HBSS (Gibco), 2 mmol/l L glucose (Sigma), 5-mg/ml HEPES (Gibco), B27 supplement (Gibco), and an antibacterial–antimycotic solution (Sigma). On the day following establishing the ex vivo culture, the serum concentration in the culture medium was gradually decreased and therefore from the 5 day in vitro (DIV) onwards, the slices were kept for the following 7 days in serum-free media and in normoxic gas conditions (5% O_2_, 5% CO_2_).

### Primary In Vitro Cultures of Rat Glial Cells

Initial primary culture comprising glial cells were isolated from 2-day-old Wistar rats bred in the Animal Care Facility (the Mossakowski Medical Research Centre). The protocol of the nervous tissue acquisition, approved by the Local Ethics Committee on Animal Care and Use, was described in detail elsewhere [52]. Briefly, the neonatal animals (*n =* 36) were put into deep hypothermia, cerebral hemispheres were isolated and mechanically dispersed in Dulbecco’s Modified Eagle’s Medium (DMEM) (Gibco supplemented with 10% fetal bovine serum (FBS, Gibco) and 1% antibiotic–antimycotic solution (Sigma, by means of a Pasteur pipette and a 1.2-mm Luer-Lock needle. Cells were then filtered through a 40.0-μm pore cell strainer (pluriSelect and seeded into 75-cm^2^ culture flasks (NUNC). After being cultured for the following 11–13 days, the subsequent fractions of glial cells were separated by gentle detaching by use of a horizontal orbital shaker (Biosan). The first detached fraction obtained due to 1-h long shaking at 160 rpm corresponded to the microglial cells which were immediately plated at density of 4 × 10^4^/cm^2^ at 6-well plates coated with poly-l-lysine (Sigma). Shaking the remaining mixed glial culture for additional 15–20 h allowed the isolation of the OPCs, which were plated at density of 1.5 × 10^4^/cm^2^. Next, the astroglial fraction was detached by mild trypsinization (5 min. at 37 °C) and after being washed with PBS, the astrocytes were seeded at the same density as microglial cells. The resulting glial monocultures (i.e., primary cultures of microglia, oligodendrocyte progenitors, and astrocytes, respectively) were grown in a serum-free medium in atmosphere containing 5% O_2_ and 5% CO_2_, corresponding to the physiological normoxia. Homogeneity of the glial monocultures were checked every time with application of the lineage-specific markers and estimated for not less than 98.5% for each of the particular subpopulation of glial cells.

### Temporal Oxygen-Glucose Deprivation as an In Vitro Model of Perinatal Asphyxia

To mimic hypoxic–ischemic insult associated with perinatal asphyxia, organotypic hippocampal slices, as well as all glial monocultures were subjected to a temporal limitation of oxygen and glucose supply (OGD: oxygen and glucose deprivation). The procedure was performed on primary cultures 24 h after plating microglia and astrocytes, 4 h after plating OPCs and on 7 DIV in ex vivo cultures of the nervous tissue slices. To retain the buffer osmotic concentration unchanged, the glucose was replaced with 10-mM mannitol. In order to eliminate oxygen, the Ringer solution used to perform OGD procedure was saturated with 95% N_2_ and 5% CO_2_. Duration of OGD in oxygen-free chamber was established for 50 min. Immediately after the OGD, the cultures were returned to their standard conditions.

### Quantitative Determination of IGF-1 Content in Primary Cell Cultures, Hippocampal Slices and Brain Hemispheres

IGF-1 quantities were measured in the samples collected from all of the in vivo, ex vivo, and in vitro models of perinatal asphyxia (Fig. 1). Accordingly, rat brains subjected to neonatal hypoxic-ischemic injury were extracted either on 1, 3, 7, or 14 day postinjury (PI) and divided into ipsilateral (to the insult) and contralateral hemispheres. Thus the applied animal model allowed us to track the impact of both the reduced blood flow and hypoxia on the nerve tissue in ipsilateral hemisphere (ipsi) and the impact of hypoxia in contralateral brain hemisphere (contra). Amounts of IGF-1 were measured in tissue lysates of cerebral hemispheres and hippocampus separately, from both the injured and the sham-operated animals. For the purpose of ex vivo studies, organotypic hippocampal slices were collected 1, 3, 5, and 7 days after OGD. Likewise, supernatants conditioned by in vitro monocultures of glial cells were collected at time points corresponding to 1 day and 3 days after OGD. For the quantification of the intracellular IGF-1, oligodendrocytes were lysed either 1 h, 1 day, or 3 days after OGD. Homogenates of the collected tissue and cell samples were prepared with the application of CelLytic solution (Sigma), supplemented with Protease Inhibitor Cocktail (1:100, Sigma). Measurements of the total protein concentrations in the lysates obtained from brain and slices were performed by means of DC protein assay (Bio-Rad), based on the standard Lowry method. For precisely measuring the lower protein concentrations (in culture supernatants and in lysates of cell cultures), Bradford Reagent (Sigma) was used. To precisely quantify the IGF-1 amounts, the ultrasensitive sandwich ELISA (enzyme-linked immunosorbent assay) was used according to the manufacturer’s recommendations (Thermo Fisher Scientific). The intensity of the resulting colorimetric reaction was measured at 450-nm wave length with use of the spectrophotometric plate reader Fluorostar Omega (BMG LabTech).

### Testing the Influence of IGF-1 on Cell In Vitro and Ex Vivo Cultures

To evaluate the influence of IGF-1 on survival, proliferation, and maturation of rat oligodendrocytes, different concentrations (varying from 10 to 50 ng/ml) of this compound (Thermo Fisher Scientific) were added to the culture medium of oligodendrocytes and the organotypic hippocampal slices immediately after OGD for the following 5 days. In another experimental variant, the endogenous IGF-1 released by glial cells to the culture medium was neutralized by addition of the extensive amounts of either antiIGF-1 (1:1000; MerckMillipore) or antiIGF-1R (receptor) antibodies (1:400; Abcam). The effectiveness of IGF-1R or IGF-1 neutralization was verified by application of ELISA method. Accordingly, the culture media were collected and assayed for testing the possible IGF-1 presence. Standard culture media (with no supplements), conditioned by control primary glial monocultures, served as a positive control. Then the cells and slices were fixed with 4% paraformaldehyde (PFA) in PBS for 20 min and 40 min, respectively, rinsed three times with PBS and subjected to immunostaining with the selected antibodies.

### Immunostaining of Differentiating Cells

In order to use immunostaining techniques, the glial monocultures were fixed 5 days after OGD, while hippocampal slices were fixed 7 days after the procedure. To verify the homogeneity of glial cell cultures, cells were labeled with the selected lineage-specific antibodies, i.e., with antiIBA1 (1:200, Abcam) to distinguish microglia, antiCNPase (1:100, Merck Millipore) to detect oligodendrocytes, and antiGFAP (1:200, Merck Millipore) to visualize astrocytes. To evaluate differentiation of oligodendrocytes in the primary cultures and in the organotypic hippocampal slices, an additional immunolabeling was performed with oligodendroglial lineage-specific antibodies directed against NG2 (1:100, Chemicon) and Olig2 (1:500, Merck Millipore). Proliferating cells were visualized with antiKi67 antibody (1:100, Leica). Unspecific binding of antibodies was eliminated by incubating the fixed cells and the slices with 10% normal goat serum (Sigma) in PBS containing 0.1% (0.25% for tissue slices) Triton X-100 (Serva) for 1 h at room temperature. All of the primary antibodies were applied overnight at 4 °C. Secondary antibodies conjugated to fluorescent dyes, i.e., Alexa Fluor-488 and Alexa Fluor-546 (1:1000, Thermo Fisher Scientific) were used to label the immunostained cells. To visualize cell nuclei, Hoechst 33342 was applied during 15 min incubation. After immersing in the Fluoromount™ reagent (Sigma), the resulting slides were used for picture acquisition by means of the LSM 780/ ELYRA PS.1 superresolution confocal system (Carl Zeiss). The analyzed area of the hippocampal slice or cell culture corresponded to 0.386 mm^2^.

### Sholl Analysis of Cell Branching

In order to evaluate a progress in the oligodendrocyte differentiation process, the branching of immunolabeled NG2^+^ cells was examined in hippocampal slices by application of Sholl morphometric analysis. Accordingly, the Z-stack microscopic images were collected, and the maximum intensity projections of individual cells were created to obtain detailed images of cells with all their branches on 2D plane. Subsequently, masks of cells were drawn with use of semiautomatic tracing method in the NeuronJ plugin to ImageJ software in order to generate the binary images of cell branching. A number of intersections of cell proccesses with consecutive concentric circles around the cell body were recorded, and a number of quantitative descriptors were calculated. All of the performed Sholl measurements were based on the following parameters: starting radius − 5 μm and radius step − 1 μm.

### Statistical Analysis

Biochemical measurements were done at least in triplicate, with two dilutions of the examined sample on each plate. The cells labeled by means of immunofluorescent techniques were counted on randomly selected 5–10 visual fields on each of at least five slides from each of the three experiments. A statistical analysis of the collected data was performed with the application of the GraphPad PRISM 8.0 software, selecting an one-way analysis of variance (ANOVA) followed by the Bonferroni’s multiple comparison test as a tool to compare experimental groups. All the data were expressed as mean standard deviation. The calculated differences were recognized as the significant if: *p < 0.05, **p < 0.01; ***p < 0.001, ****p < 0.0001.

## Results

### Influence of Hypoxic-Ischemic Insult on Endogenous IGF-1 Expression in Rat Neonatal Brains

**Fig. 2:**
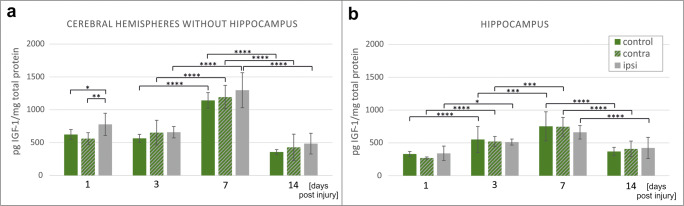
IGF-1 level in rat brains at various time-points after hypoxic-ischemic insult applied to P7 rat pups, determined in: **a** Brain hemispheres (without hippocampus) and **b** Isolated hippocampal region. The plain green bars represent concentration of IGF-1 in brains of control animals, striped green bars represent hemispheres contralateral to the site of the injury (which became hypoxic due to the applied model) and the gray bars corresponds to the hemispheres ipsilateral to the site of injury (hypoxic-ischemic). Presented values are mean ± standard deviation. The differences between the examined groups were marked as statistically significant if: significant*p < 0.05, **p < 0.01; ***p < 0.001, ****p < 0.0001

With aim of addressing the question about the potential impact of neonatal hypoxia-ischemia on the IGF-1 availability in the affected brains, the animal in vivo model of perinatal asphyxia was used (Fig. 1). Taking into consideration that the main neurological disabilities resulting from asphyxic injury in survived babies might be associated with the malfunctioning of hippocampus, this region was isolated and analyzed separately. Additionally, to evaluate the possible influence of HI on the hemisphere contralateral to the insult, the IGF-1 levels were measured separately in the both brain hemispheres (Fig. 2a) and the both hippocampi (Fig. 2b). Accordingly, as revealed by quantitative studies, just 1 day after the HI insult (1 PI), the amount of IGF-1 was significantly elevated in the ipsilateral hemisphere in comparison with the hemisphere contralateral in relation to the insult (38.7%; *p <* 0.01) and to control brains (24.9%; *p <* 0.05) (Fig. 2a). The examination of tissue lysates obtained from the brains collected 3, 7, and 14 after the injury did not revealed any statistically relevant differences between the injured and the control hemispheres (*p >* 0.05). This suggests that during the later post injury period, the amounts of IGF-1 decreased and stabilized at the physiological level. Interestingly, the highest endogenous level of IGF-1 in the neonatal brains was detected 7 days after the injury (which corresponds to P14) and was estimated at 1143.00 ± 119.37 pg per mg of the total protein content. During the following week (7–14 PI), an approximate 3-fold decrease (to 357.94 ± 35.74 pg/mg total protein content) was recorded in the samples derived from the control brains (Fig. 2a). While examining hippocampi, a 2-fold decrease in the IGF-1 level was observed between day 7 and 14 postinjury (from 752.53 ± 221.73 to 368.73 ± 64.39 pg IGF-1/mg of total protein content) (Fig. 2b). Interestingly, the upregulation of the IGF-1 level 24 h after the HI insult was not observed in this brain region. However, IGF-1 quantification at different time-points postinjury indicates that the observed increase in its physiological amounts proceeds more slowly in the hippocampi affected by HI (*p <* 0.05 for 1PI vs 3PI and *p >* 0.05 for 3PI vs 7PI) in comparison with controls (*p* < 0.0001 for 1PI vs 3PI and *p* < 0.001 in case of 3PI vs 7PI).

### Influence of Hypoxic-Ischemic Insult on Paracrine/Autocrine IGF-1 Release in Ex Vivo Cultures of Organotypic Hippocampal Slices

**Fig. 3:**
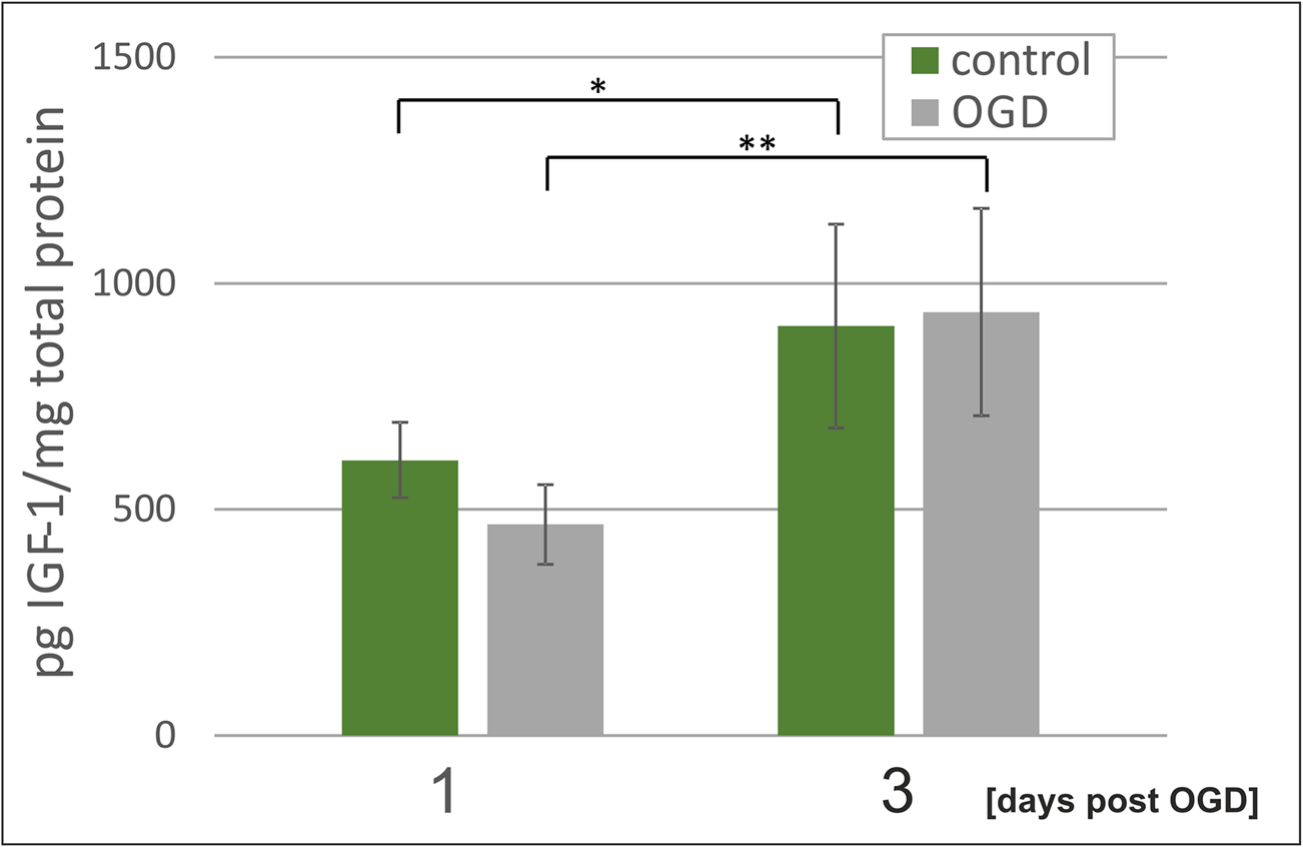
IGF-1 quantification in the organotypic hippocampal slice culture 1 day and 3 days after oxygen-glucose deprivation. The concentrations of IGF-1determined by ELISA-based measurement were normalized versus total protein concentration in individual samples. Presented values are mean ± standard deviation. *p < 0.05, **p < 0.01

To eliminate potential augmentation of centrally generated IGF-1 and delivered via brain-blood barrier (BBB) to the nervous tissue, the ex vivo cultures of organotypic hippocampal slices were used. This approach (presented in Fig. 1) allowed us to focus on the paracrine/autocrine effects of IGF-1 secreted in situ by neural cells. As revealed by the obtained data, the IGF-1 amounts in the hippocampal slices cultured up to 14 days (7 days before and 7 days after OGD) were similar to those determined in the hippocampi during the in vivo studies, pointing to the important role of neural cells as the endogenous source of this factor in hippocampal region (Fig. 3). Likewise, there was also no difference between the control and the OGD-treated tissue. The amount of IGF-1 in the samples collected 5 days and 7 days after the insult was below the lower limit of detection defined by the ELISA kit’s manufacturer, suggesting decrease in IGF-1 secretion during following days of the ex vivo culture. Since cell differentiation and senescence is remarkably accelerated in the cultured tissue slices, this time-point seems to be relevant to the 7–14 PI period of the in vivo model.

### Effect of IGF-1 Supplementation on Oligodendroglial Proliferation in Hippocampal Slices

**Fig. 4:**
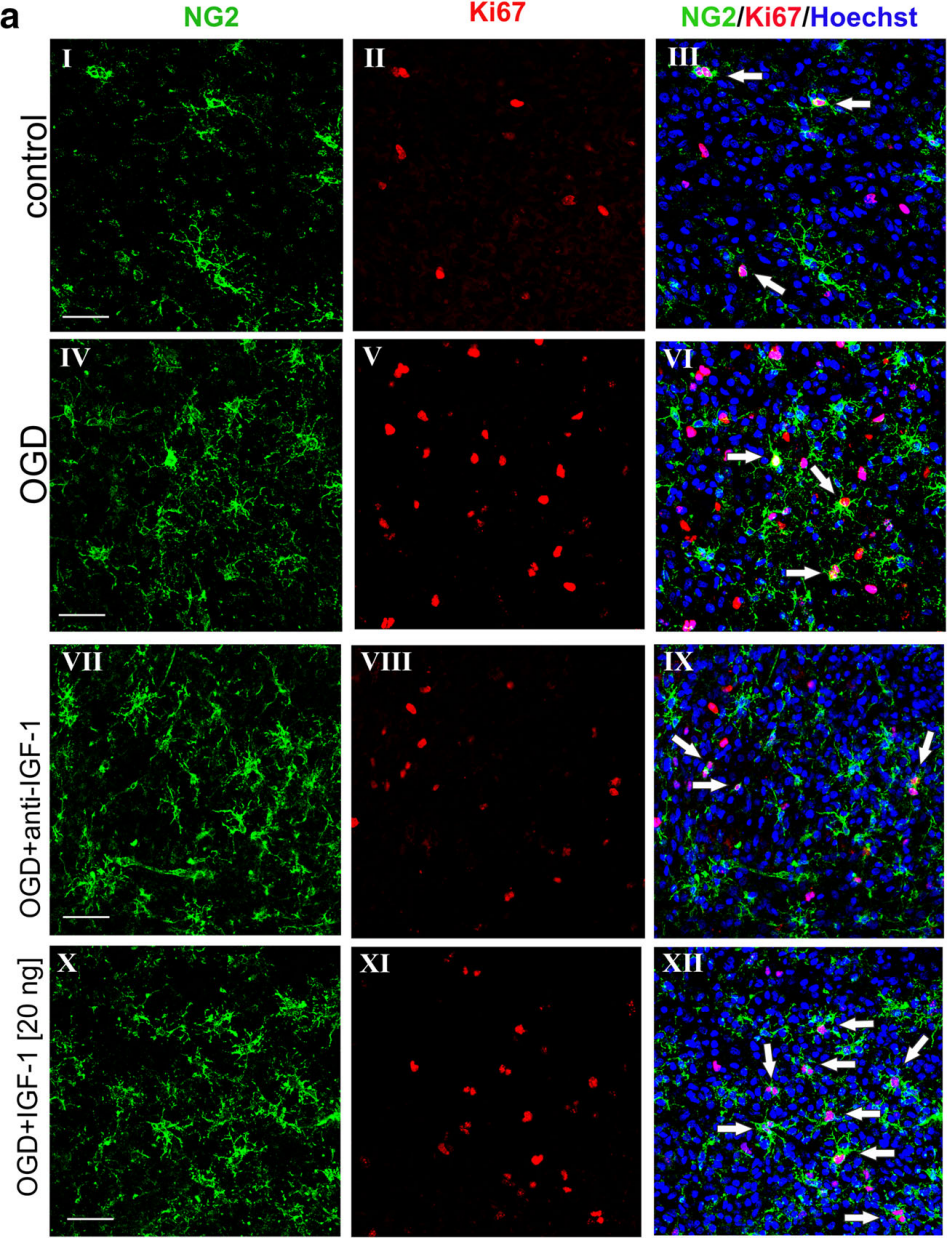

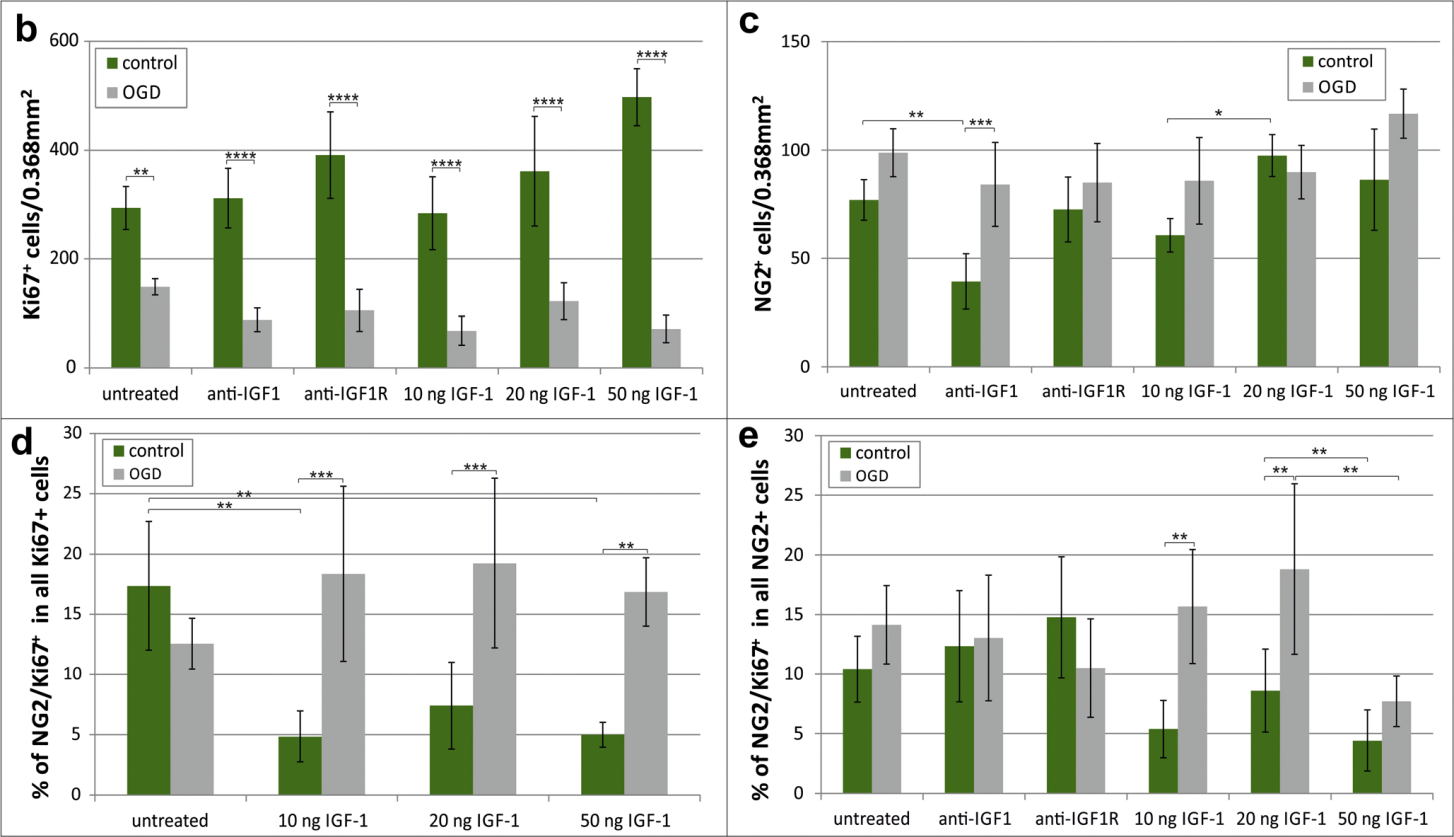
Impact of oxygen and glucose deprivation followed by various treatments on proliferation of neural cells in the organotypic hippocampal slices, determined 5 days after the insult. **a** Immunohistochemical visualization of oligodendrocyte progenitors (NG2^+^, green) and proliferating, Ki67-positive cells (red). Cell nuclei are stained with Hoechst 33342 (blue). White arrows indicate dividing oligodendroglial progenitors (NG^+^/Ki67^+)^. Microphotographs show hippocampal slices cultured in control conditions (I-III), affected by OGD (IV-VI) and after different treatments applied to OGD-injured cultures (VII-XII). Scale bar is equivalent of 50 μm. **b** Evaluation of the number of dividing neural cells after OGD and in response to various experimental treatment; **c** The number (expressed as the absolute values) of OPCs in various experimental conditions; **d** Calculation of the dividing OPC fraction in the entire pool of cycling cells in the nervous tissue of the hippocampal region. **e** Increase in number of proliferating OPCs in OGD-injured hippocampal slices treated with various IGF-1 doses. Presented values are mean ± standard deviation; the results were considered as significant if: *p < 0.05, **p < 0.01; ***p < 0.001, ****p < 0.0001

To identify the proliferating cells within the nervous tissue of the hippocampal region, the antibody against Ki67 protein in the dividing cell nuclei together with markers specific for oligodendroglial lineage were used. First of all, the possible influence of various IGF-1 doses on oligodendrocyte progenitor cells was tested. As determined by quantitative biochemical methods, the IGF-1 concentrations range between 0.5 and 1 ng per mg of total protein content in the nervous tissue (Figs. 2 and 3). Since in the performed ex vivo study the cultures of hippocampal slices were supplemented with the exogenous IGF-1, the calculation of concentrations of the added compound were based on the manufacturer recommendations. Likewise, supplementing oligodendroglial or slice cultures with insulin (which is an analog of IGF-1) in concentration of 10 ng/ml (for instance in ITS supplement) was recommended for use to support oligodendroglial survival. Therefore, the tested IGF-1 doses were estimated for 10, 20, and 50 ng per ml of culture media, and their influence on oligodendrocyte proliferation was verified by immunohistochemical analyses. The first interesting observation concerned the significantly (*p <* 0.01) reduced number of dividing neural cells in hippocampal slices after OGD insult when compared with untreated controls (Fig. 4a, b). While added at the highest of the applied doses (50 ng/ml), the IGF-1 turned out to stimulate the process of cell proliferation in the control slices (*p <* 0.05) when compared with those untreated with IGF-1; however, the given treatments seemed to be insufficient to promote cell division in the injured slices and to stimulate in this way process of neuroregeneration. Accordingly, the cell proliferation rate in OGD-injured slices cultured with addition of IGF-1 at concentration of 50 ng/ml was still significantly (*p* < 0.0001) lower than in control slices supplemented with the same IGF-1 dose (Fig. 4b).

Focusing the analyses on oligodendroglial progenitor cells, visualized by immunolabeling the membrane marker NG2, specific for the very early stage of oligodendrocyte differentiation process, also showed no significant effect on enhancing rate of cell proliferation after treatment with the applied doses of IGF-1. Interestingly, in spite of being temporarily deprived of oxygen and glucose, the number of proliferating OPCs in the entire fraction of the dividing neural cells remained markedly unchanged when examined 5 days after the insult. Accordingly, in the cultured hippocampal slices of rat neonatal brain, the dividing OPCs (NG2^+^/Ki67^+^) constituted 17.35 ± 5.33% in controls and 12.55 ± 2.11% after the OGD procedure of all the cycling cells (Fig. 4c). Subsequent examination of the results of the supplementing slice cultures with different IGF-1 doses indicated a general, significant decrease of the NG2^+^/Ki67^+^ fraction in control slices, while after OGD this proportion significantly increased (Fig. 4d). This was further confirmed by examination of the number of dividing OPCs within total NG2^+^ fraction. Accordingly, the performed statistical analysis revealed that the stimulation of OPC proliferation by was most pronounced when IGF-1 was added at concentrations of in the injured slices is the most pronounced in concentration of 10–20 ng/mg (*p <* 0.01) (Fig. 4e). The obtained results showed that IGF-1 specifically increases proliferation rate of oligodendroglial precursors after OGD injury.

**Fig. 5:**
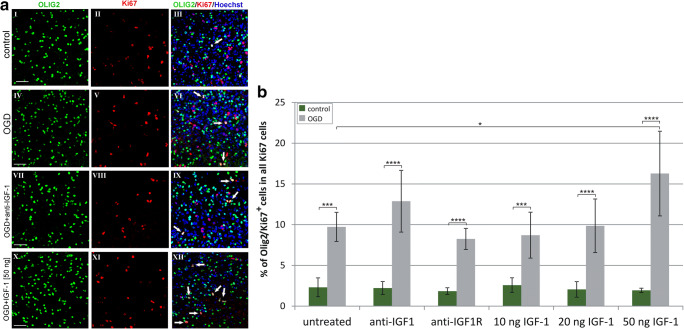
Impact of oxygen and glucose deprivation followed by various treatments on proliferation of oligodendroglial cells (labeled with the lineage-specific marker Olig2) in the organotypic hippocampal slices, determined 5 days after the insult. **a** Immunolabeled oligodendrocytes (Olig2^+^, green) and proliferating cells visualized by anti-Ki67 antibody (red). Cell nuclei are stained with Hoechst 33342 (blue). White arrows indicate dividing cell nuclei in oligodendrocytes (Olig2^+^/Ki67^+^). Microphotographs show hippocampal slices cultured in control conditions (I-III), affected by OGD (IV-VI) and after different treatments of OGD-injured cultures (VII–XII). Scale bar is equivalent of 50 μm. **b** Calculation of the dividing oligodendroglial fraction in the entire pool of cycling cells. The values are mean ± standard deviation; the statistical differences were considered as significant if: *p < 0.05, ***p < 0.001, ****p < 0.0001

The subsequent examination of Olig2 immunolabeling, an oligodendroglial lineage-specific transcription factor expressed during both the cell commitment and differentiation, indicated however that the injury stimulated the oligodendrocytes to propagate (Fig. 5a, b), causing a 67.3% increase in the number of Olig2^+^/Ki67^+^ cells when the slices were treated with 50 ng IGF-1 after OGD (*p* < 0.05). As shown, neither neutralizing IGF-1 nor blocking its receptor had an impact on the number of Olig2-expressing cells in the hippocampal slices (Fig. 5b). This observation suggests that in the selected concentrations, IGF-1 might be effective in promoting oligodendroglial proliferation, which is not limited however to the progenitor stage only.

### Supplementation of IGF-1 Stimulates Ramification of Oligodendroglial Processes in Hippocampal Slices

**Fig. 6:**
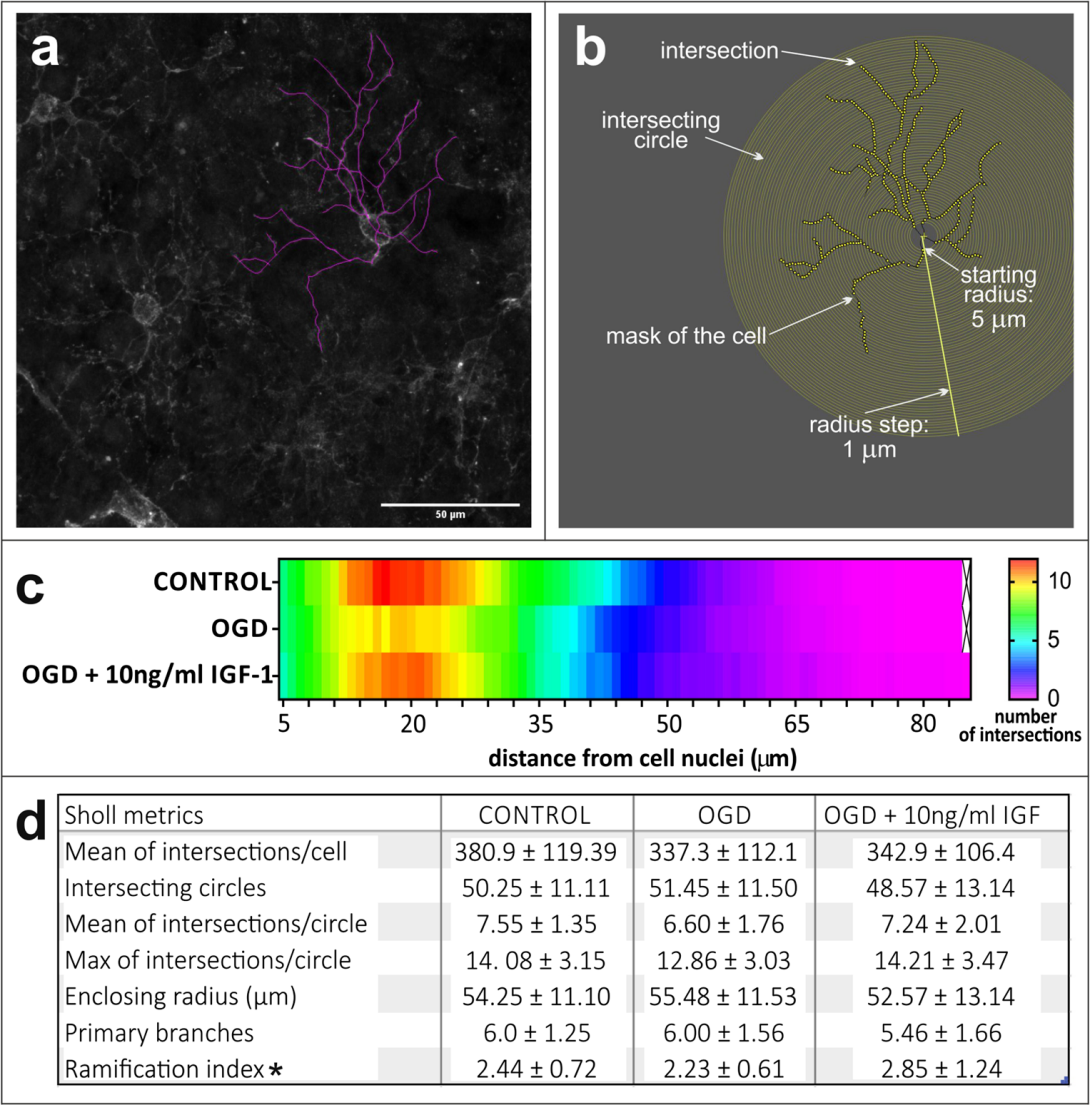
Morphometric analysis of the branched, NG2-positive oligodendrocytes in the organotypic hippocampal slices. The graphical masks of an individual cell were drawn by application of NeuronJ software (**a**) and then subjected to Sholl analysis (**b**). Number of intersections of cell branches with consecutive concentric circles around cell body were recorded (**c**), and a number of quantitative descriptors was calculated (**d**). The differences were recognized as significant if *p < 0.05

With regard to the fact that one of the descriptors of oligodendrocyte differentiation is the complexity of the elaborated cell processes, a morphometric examination of the multibranched NG2-positive immature oligodendrocytes was performed. Accordingly, the labeled cells grown in the lowest, 10 ng/mg IGF-1 concentrations, were subjected to the Sholl analysis (Fig. 6a, b).

As shown by the linear plot (Fig. 6c), the control cells were the most branched, in spite that the number of primary branches and enclosing radius (indicating the size of the cell) were similar to the parameters recorded for the cells injured by the OGD procedure. Additionally, among numerous descriptors calculated for the relevant images (Fig. 6d), the ramification index (corresponding to the ratio of the maximum number of intersections to the number of primary branches) pointed to the most interesting differences between the examined variants of experimental work. Namely, statistically significant differences (*p <* 0.05) were observed when the hippocampal slices subjected to the OGD procedure were compared with those treated with 10 ng/ml IGF-1 after the insult. This indicated that supplementing the culture media after OGD with IGF-1 in as low concentrations as 10 ng/ml led increasing the number of intersections in the cell processes, thus promoting their ramification and indicating the role of this growth factor in oligodendrocyte differentiation.

### IGF-1 Secretion by Neonatal Glial Cells Is Differently Affected by Hypoxic-Ischemic Condition In Vitro

To address the question if any type of glia may be responsible for the observed changes in the endogenous IGF-1 content at 24 h after the applied insult, the secretion of this factor was evaluated in the obtained glial monocultures. As revealed by the quantitative biochemical studies, an autocrine expression of IGF-1 strictly depended on the type of glial cell. Accordingly, the level of IGF-1 measured in culture media conditioned by astrocytes was a whole order of magnitude higher than those enriched by IGF-1 released by either microglia or differentiating oligodendrocytes.

**Fig.7:**
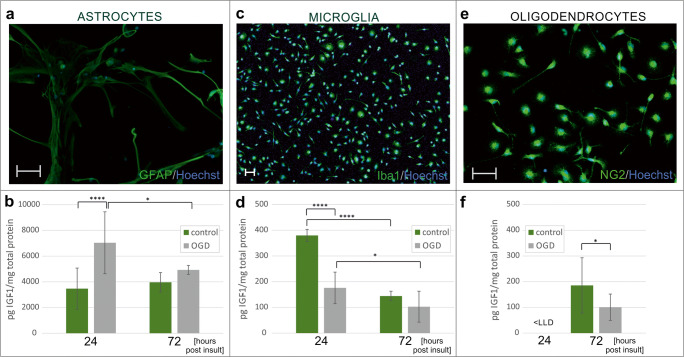
Quantification of IGF-1 in the culture supernatants of astrocytes (**a**, **b**), microglia (**c**, **d**) and oligodendrocytes (**e**, **f**) 24 and 72 h after oxygen-glucose deprivation, respectively. Monocultures are stained with the lineage-specific markers (green), while cell nuclei are visualized with Hoechst 33342 (blue). Scale bars corresponds to 50 μm. Presented values are mean ± standard deviation *p < 0.05, ****p < 0.0001

While IGF-1 secretion by astrocytes was 1.5-fold increased during the first day after OGD when compared with control values (*p <* 0.0001; Fig. 7a, b), the amounts of this growth factor released by microglia is 2 times lower than in control culture (1 day after OGD; *p <* 0.0001; Fig. 7c, d). Similar effect was observed during examination of oligodendrocyte cultures (3 days after OGD; *p* < 0.05; Fig. 7e, f). Concentration of IGF-1 in media collected from oligodendrocyte culture at 24 h after OGD was below the lower limit of detection; therefore, the additional measurements were performed in cell lysates. The mean amount of the intracellular IGF-1 in controls was estimated at 859.03 ± 206.47 pg/mg total protein content),while after 1 h after OGD, it was reduced to 765.81 ± 192.45 pg/mg total protein (*p =* 0.4).

### Supplementing Oligodendroglial Cultures with Exogenous IGF-1 Does Not Affect Cell Proliferation but Promotes Their Differentiation

**Fig.8:**
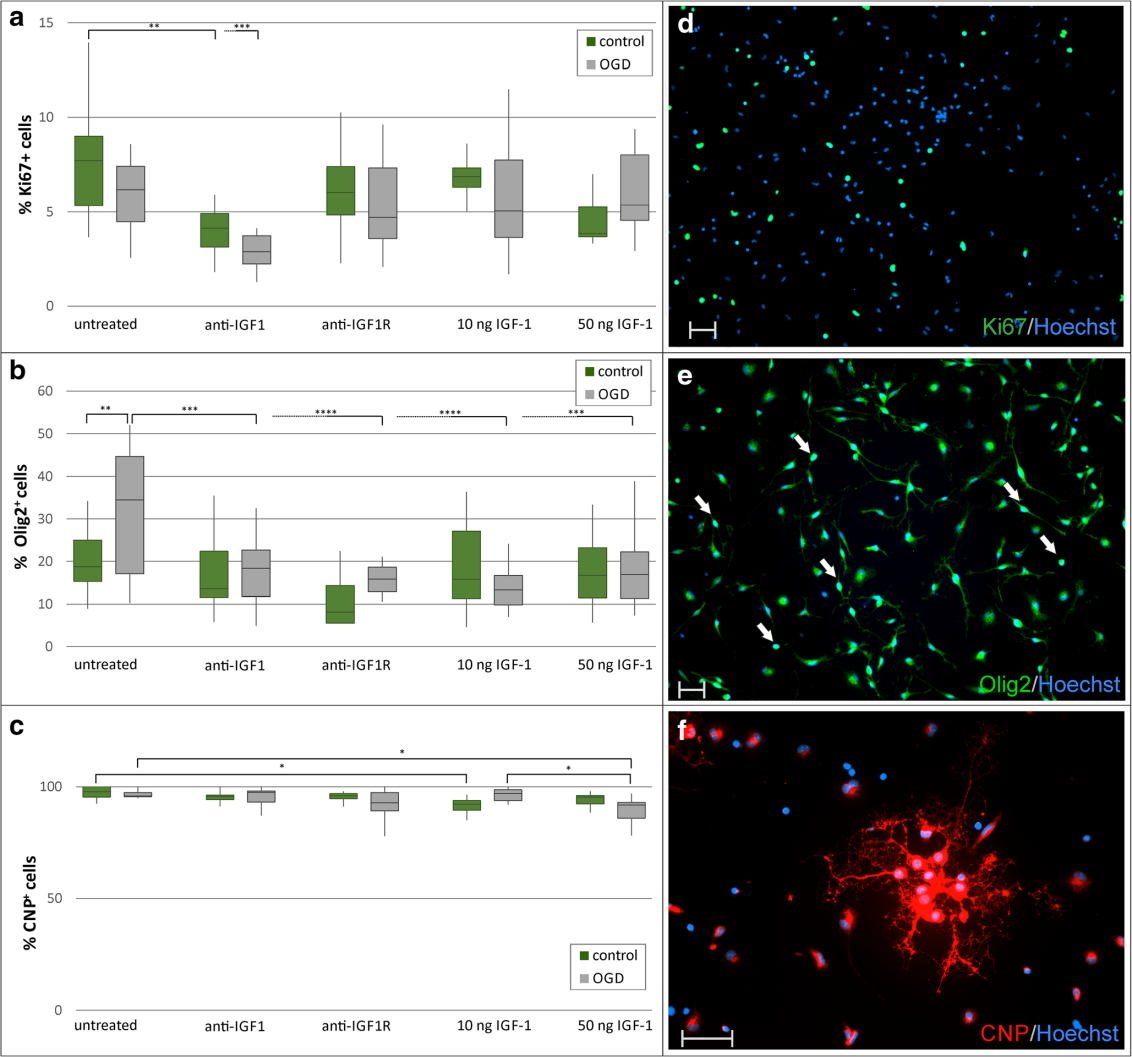
Immunocytochemical analysis of oligodendrocyte cultures fixed 5 days after OGD followed by testing various experimental treatments. The applied antibodies included anti-Ki67 to label proliferating cells (**a**, **b**), as well as anti-Olig2 to stain transcription factor localized in the cell nuclei (**c**, **d**) and antiCNPase (**e**, **f**) to visualize differentiating oligodendrocytes. Results are showed as box and whisker graphs. A vertical line in box represents a median of the obtained values. Bottom and upper edges of the box represent the first and third quartile, respectively. Whiskers indicate the minimum and the maximum value of the results. Scale bar equals 50 μm

Results obtained during studies on hippocampal slices were verified by establishing primary cultures of rat oligodendrocyte progenitors, performing OGD procedure to evoke HI stress and applying the same treatments as for the cultured hippocampal slices. Likewise, the opposite experimental approaches were used. The first one was based on eliminating the IGF-1 influence by either neutralizing this factor or blocking its receptor with specific antibodies. The second approach involved supplementing culture media immediately after the injury with rat recombinant IGF-1 at concentrations 10 ng/ml and 50 ng/ml. To evaluate the results of the applied treatments on proliferation and differentiation of cultured oligodendrocytes, the expression of the selected lineage specific markers was examined by means of immunocytochemistry 5 days after OGD injury. Accordingly, as deduced from calculation of the immunolabeled Ki67-positive cells, addition of IGF-1 immediately after OGD did not stimulated oligodendrocyte proliferation (Fig. 8a, b). However, neutralization of oligodendrocyte-derived IGF-1 resulted in a significant (*p <* 0.01) decrease of the number of dividing cells (Fig. 8b), suggesting an autocrine effect on the cell proliferation.

The obtained results also showed that applying any type of the designed treatments after OGD resulted in a significant decrease in fraction of Olig2^+^ positive cells, as deduced from estimating number of cells in which this transcription factor was localized in the cell nuclei (Fig. 8c, d). Subsequent analysis of differentiating, CNP-positive oligodendrocytes, characterized by more complex morphology revealed that IGF-1 concentration of 50 ng/mlin culture media after OGD reduced the number of maturing cells (to 89.9% ± 5.9% of CNPase^+^ cells), when compared with either OGD-subjected and untreated cell cultures (96.6% ± 1.9% of CNPase^+^ cells; *p* < 0.05) or to the OGD-subjected cultures supplemented with IGF-1 at concentration of 10 ng/ml (96.3% ± 2.7% CNPase^+^ cells; *p* < 0.05; Fig. 8e, f). Those results point to the important role of the local IGF-1 concentration in regulation of oligodendrocyte maturation.

## Discussion

The cells forming the nervous tissue are functionally interdependent and interplay by both responding to and releasing numerous factors, which altogether create the local tissue microenvironment. This is especially pronounced in the case of gliogenesis process and oligodendrocyte differentiation, in which the subsequent developmental stages are strictly regulated by the in situ concentration of instructive signals [4, 52–55]. Among them, the IGF-1 is thought to be one of the most important factors engaged in regulation of the majority of processes associated with oligodendrocyte functioning.

The growing list of evidence points to the altered availability of IGF-1 in a wide spectrum of neurodegenerative disorders. While after traumatic brain injury (TBI) the endogenous IGF-1 level has been shown to increase, in multiple sclerosis (MS), which is one of the most deeply studied demyelinating diseases, a local deficiency of this growth factor has been observed, often within the areas of demyelination. Alteration of IGF-1 level is thought to be also one of the features of the white matter disorders developing in a result of the experienced perinatal asphyxia. Since the neonatal period coincidences with the most active processes of gliogenesis, oligodendrocyte survival and differentiation, as well as myelination of the newly derived neurons, the insufficient amounts of endogenous trophic factors, including IGF-1, might unfortunately efficiently contribute to the resulting myelin deficiency, or/and malformation of the white matter tracts [56–59]. The recent clinical studies confirmed that the IGF-1 level in serum is significantly decreased in newborns with hypoxic-ischemic encephalopathy [60]. A number of preclinical studies have been performed based on IGF-1 systemic administration in order to at least partially alleviate deleterious effects of the experienced birth asphyxia. Depending on the way and mode of the IGF-1 administration (for instance intraventricular versus intranasal or subcutaneous, in one or several repeated doses, within narrow therapeutic window or after the onset of first neurological symptoms, alone, or combined with other therapy etc.), different beneficial effects have been reported in animal models of birth asphyxia. Among others, increased cell proliferation, rescued loss of Olig2-positive cells in the white matter and reduced lesion volume were observed [61–64]. To date however, the moderate therapeutic hypothermia is the sole clinical intervention after perinatal asphyxia. Moreover, even if applied within the narrow therapeutic window and for a prolonged time, hypothermia does not always confer neuroprotection and has been shown to be unsuccessful in more severe cases of asphyxic episodes. Actually, it seems that hypothermia neither provides complete brain protection nor stimulates the repair necessary for improving the neurodevelopmental outcome. New treatment options are therefore urgently needed to protect fragile developing brains from the fatal consequences of hypoxic–ischemic damage and to restore their proper physiological functioning [65–67].

Recently, there is an ever growing recognition concerning the role of autocrine/paracrine IGF-1 signaling in brain development and metabolism [68, 69]. This factor is known to be distributed by the circulating blood system due to endocrine production under control of growth hormone (GF) and actively transported to the CNS through the choroid plexus by transcytosis; however, it is also secreted in situ by various cell types [70, 71]. Recent findings highlight the role of cells inhabiting the nervous tissue as a source of locally available IGF-1 in tissue microenvironment. It is hypothesized the endogenous IGF-1 released in situ by neural cells exerts the main pleiotropic effects on the neighboring cell functions.

To address the issue about the role of central versus paracrine IGF-1 secretion in oligogliogenesis, a study based on a step-wise approach was performed. First of all, the endogenous quantities of IGF-1 were determined in the rat brains after the insult triggered by the transient hypoxic-ischemic conditions. After considering the timeline of neonatal brain development, especially in the context of progress in myelination of various brain regions in humans versus rodents, P7 rats, which correspond to 30th postconceptional day (PC), were selected for performing the animal model of perinatal asphyxia [72–74]. Accordingly, 7th postnatal day in rats is still within the peak on oligogliogenesis and just prior to the onset of myelination in the hippocampus (33 PC), striatum (34 PC), and corpus callosum (35 PC). These facts seemed to be of vital importance in the context of our previous findings which showed that myelination in the indicated brain regions 10 weeks after the HI was significantly deficient or aberrant [75]. Apart from the existence of the malformed myelin sheaths in the hippocampus, the data obtained in another study showed the impaired processes of neurogenesis and gliogenesis in the rat hippocampus in the in vivo model of perinatal asphyxia [76]. Taking into consideration the above mentioned observations, together with the proven cognitive and intellectual dysfunctions being the main and commonest consequences of the experienced perinatal asphyxia [69, 77–81], the hippocampal region was subjected to close (i.e., in vivo and ex vivo) scrutiny.

The applied approach led to interesting observations about the significant, transient increase of the IGF-1 amounts in neonatal brains 24 h after the insult. However, the similar effect was absent in the examined hippocampi. To further evaluate, whether the increased IGF-1 quantities are derived from the central sources and are due to the intensified penetration of this factor via neonatal BBB, the subsequent in-depth studies were based on series of ex vivo experiments, which allowed eliminating the influence of the peripheral IGF-1, distributed by the circulatory system. This approach confirmed the previous in vivo observations concerning the unaltered IGF-1 level in the nervous tissue localized within the hippocampal regions. However, in spite of the lack of alterations in the IGF-1 amounts, a decreased rate of cell proliferation was observed within the ex vivo examined hippocampal area. Interestingly, the proportion of the dividing OPCs to the rest of the cycling neural cells remained unchanged which was due to the observed accelerated proliferation of oligodendroglial progenitors.

This new observation could be contributed to the application of physiologically relevant normoxic conditions for the entire performed study. As revealed by the data obtained from one of our recent studies [51], oxygen level is one of the major factors affecting proliferation and differentiation of oligodendrocytes. To date, the majority of the published studies on glial cells were conducted in standard laboratory conditions, i.e., the cells were cultured in ambient (corresponding to 16–21%) oxygen concentration, which however was shown to accelerate cell maturation. In the present study, the organotypic slices were cultured after OGD in conditions corresponding to physiological normoxia, i.e., in 5% O_2_, relevant to physiological conditions found in the nervous tissue [82–84]. The period of cell proliferation was therefore extended, and the deficiency of oligodendrocyte progenitors was supposedly compensated for in response to the insult. Additionally, due to testing various doses of IGF-1, the composition of the culture medium was seriously restricted. Namely, ITS supplement, which is routinely added to cell and slice cultures, was not used during the present study, due to its containing insulin, transferrin, and selenium. Adding transferrin is also supposed to accelerate oligodendroglial differentiation [85]. Transferrin might also induce cell death by ferroptosis [86]. Taken together, the process of oligodendroglial maturation in the presently created conditions seems to have slowed-down in comparison with the previous observations and thus more resembling physiological proceeding of the examined process. Accordingly, as shown by the present study, although IGF-1 levels at macroscopic examinations seemed to be unaltered, the local IGF-1 concentrations, and its bioavailability due to in situ secretion by glial cells could modulate oligodendroglial functioning. Taking into consideration the IGF-1 half-life (counting for few-minutes), the local paracrine effect might be critical for normal oligodendroglial development.

**Fig. 9:**
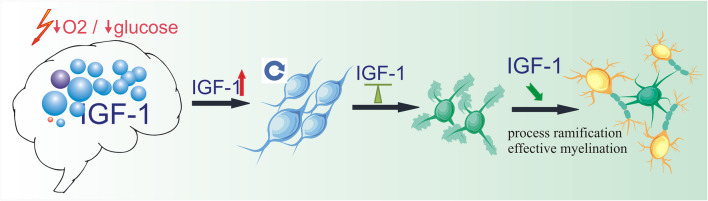
Potential therapeutic strategy aiming at promotion of oligodendrocyte maturation. Perinatal asphyxia leads to a transient increase of IGF-1 level in neonatal brain, inhibiting cell proliferation in developing brain. In the later period, IGF-1 stabilizes on the physiological level. OPCs deficiency triggered by perinatal asphyxia is compensated by cycling and differentiating oligodendrocytes. IGF-1 administration (or enhancement of IGF-1 expression by cells within the nervous tissue) would promote branching of cell processes, improving efficiency of myelination process

Likewise, with the aim of precisely evaluating the IGF-1 impact on oligodendroglial differentiation in the nervous tissue microenvironment, a morphometric analysis of the differentiating cell morphology by means of the Sholl software was applied. This allowed us to assess the degree of ramification of the cell processes, elaborated during the differentiation process to enwrap around axons and to form a myelin segment. Oligodendrocytes are known to be able to myelinate several neighboring axons; thus, subsequent myelin segments are made up by different oligodendroglial cells. From this point of view, there is no need for cells to elongate their processes in search of axons to be myelinated. Oligodendroglial response to local instructive signals, derived from neurons and triggering myelinogenesis [87–89], seems to be assured rather by high complexity of process branching. Numerous cell extensions allow sensing in situ the paracrine signals, as well as those provided by direct cellular interactions (cell-to-cell contacts). As alluded to above, the examination of several morphometric descriptors of oligodendroglial cells differentiating in the microenvironment of the nervous tissue of the hippocampal region revealed that the IGF-1 impacted significantly on the ramification of cell processes, even in the lowest of the tested IGF-1 concentrations. As therefore could be deduced from the presented ex vivo study, treatment with the tested growth factor is effective in stimulating OPC proliferation, and it might efficiently improve cell maturation. This finding is important in the context of the previously reported observations of retarded oligodendrocyte differentiation and aberrant myelinogenesis after the neonatal hypoxic-ischemic episode [75, 90]. It could also be correlated with clinical observations of the white matter disturbances in the brains of babies who survived perinatal asphyxia and associated with the following underdevelopment of enfant cognitive processes [91–94]. As therefore could be deduced from the presented studies, a transient increase in the IGF-1 level in neonatal brains during the first 24 h after hypoxic-ischemic insult leads to an inhibition of oligodendroglial proliferation, which is afterwards compensated for by the increased number of dividing cells in the later period after perinatal asphyxia, when IGF-1 stabilizes at the physiological level. It seemed to be reasonable to administrate the IGF-1 in that period as a pharmacological treatment to promote oligodendrocyte maturation and to prevent the development of white matter disorders (Fig. 9).

Those conclusions were further supported by the data obtained from the subsequent studies aimed at testing the influence of various IGF-1 doses on oligodendrocytes in vitro conditions mimicking physiological normoxia. Accordingly, an analysis carried out on CNP-ase^+^ oligodendrocytes characterized by complex morphology typical of the advanced stage of the maturation process, confirmed that IGF-1 was highly efficient in preventing the arrestment of oligodendrocyte differentiation after the hypoxic-ischemic insult. Moreover, our results indicate that neonatal oligodendrocytes are themselves a source of this factor. Contrary to astrocytes, however, hypoxic-ischemic injury negatively influences IGF-1 release by oligodendrocytes.

Among the investigated types of glial cells in the context of IGF-1 secretion, astrocytes attract a special attention. They were shown to express both IGF-1 and its receptors, which play a crucial role in regulating glucose uptake by these cells and thus providing neurons with metabolic substrates on demand [95–99]. The increased secretion of this growth factor by astrocytes exerted neuroprotective effect on hippocampal neurons after traumatic brain injury [100]. The impact of IGF-1 on the increase in number of astrocytes, as well as elevated amount of connexins and gap junctions was reported [101]. Additionally, insulin-like growth factor 1 gene therapy was shown to promote astrocyte branching [102]. Interestingly, as shown in the present study, astrocytes actively respond to temporal hypoxic-ischemic condition by significantly upregulating secretion of IGF-1 protein during first 24 h after the insult.

There is also the growing recognition of the role the microglial cells play in neonatal neurodevelopment. Namely, this cell population (CD11c^+^) exhibits unique properties during the early postnatal period, providing neurosupportive and myelinogenic signals [103, 104]. Oligodendrocyte-microglia cross-talk has been shown to play an important role both under physiological conditions (during neurodevelopment and aging) and in different pathological disorders [105–108]. Moreover, there is a growing list of evidence that IGF-1 is involved in modulation of neuroimmunological processes [109–112]. Since neuroinflammation is associated with the majority of diseases distinguished as white matter disorders and associated with hypo- or demyelination of the nervous system [113, 114], this growth factor is considered to be one of the main tools in combating the mentioned neurodegenerative disorders. Postulated involvement of IGF-1 in promoting angiogenesis [115] and in this way helping to overcome oxygen and metabolic crisis within the tissue affected by transient hypoxia-ischemia, might additionally contribute to initiation of neuroregenerative processes. In this context, the downregulation of IGF-1 expression by microglial cells, shown in the present study, may contribute not only to oligodendrocyte malfunctioning but also to in situ modulation of neuroinflammation.

Taken together, the accumulating evidences of the important influence of IGF-1 in the early development of the nervous system indicate the major role of autocrine source of this compound, essential for proliferation and maturation of neural cells and establishing cellular interactions. In our previous neurodevelopmental studies, we have shown that even subtle, physiological changes in composition of local tissue microenvironment (specific for a given brain region) are potent to drive commitment and differentiation of neonatal oligodendrocyte progenitors [54]. Local concentration of IGF-1, resulting among others from autocrine activity of macro- and microglial cell, de facto modulates in situ cell functioning. It seems to be especially important for oligodendrocytes and their progenitors, which are known to be vulnerable to different types of insults (including hypoxic-ischemic injury) and extremely sensitive to external stimuli, present either in normal or pathophysiological microenvironment. Thus fine tuning of IGF-1 availability in extracellular compartments could turn out to be crucial for completing a multistep process of oligodendrocyte differentiation, which manifests itself by acquiring ability for myelinogenesis.

Keeping in mind diversified secretory response of glial cells to hypoxia-ischemia, leading to a changed tissue homeostasis and limited effectiveness of strategies based on IGF-1 administration, potential alternative strategies to promote neuroreparative processes could be considered [116, 117]. It seems reasonable to indicate the cells forming neural tissue as a target of pharmacological interventions in pathological conditions affecting CNS. Instead of-or in addition to- supplying exogenously IGF-1, which has to cross the BBB to exert its pleiotropic effects, the medicaments (like for instance small molecules, cAMP), might be preclinically tested and used in modulating microglia response to insult (e.g., promoting polarization between M1 proinflammatory and M2 antiinflammatory phenotypes) and in this way enhance endogenous in situ IGF-1 release to the local tissue microenvironment. Such an indirect strategy, based on targeting other types of neural cells, might be beneficial for stabilizing tissue homeostasis thus improving oligodendrocyte functioning in pathophysiological conditions and contributing to restorative processes.
